# Metoprolol Improves Endothelial Function in Patients with Cardiac Syndrome X

**Published:** 2016

**Authors:** Maryam Majidinia, Yousef Rasmi, Mohammad Hassan Khadem Ansari, MirHossein Seyed-Mohammadzad, Ehsan Saboory, Alireza Shirpoor

**Affiliations:** a *Department of Biochemistry, Faculty of Medicine, Urmia University of Medical Sciences, Urmia, Iran.*; b *Cellular and Molecular Research Center, Urmia University of Medical Sciences,Urmia, Iran. *; c *Department of Cardiology, Faculty of Medicine, Urmia University of Medical Sciences, Urmia, Iran. *; d *Nurophysiology Research Center, Urmia University of Medical Sciences, Urmia, Iran. *; e *Department of Physiology, Faculty of Medicine, Urmia University of Medical Sciences, Urmia, Iran.*

**Keywords:** Metoprolol, Endothelial function, Cardiac syndrome X, Angiography, Nitric oxide

## Abstract

Endothelial dysfunction which is manifested by the loss of nitric oxide bioavailability, is an increasingly recognized cause of cardiac syndrome X (CSX) and beta blockers are used for the treatment of this syndrome. Thus, the aim of this study was to investigate effects of metoprolol, as a beta blocker, on endothelial function in CSX patients.

The study included 25 CSX patients (20 female/ 5 male, mean age: 55.36±10.31 years) who received metoprolol (50 mg BID) for one month. In addition, 25 healthy controls (20 female/ 5 male, mean age: 54.32 ±9.27 years) were enrolled. Levels of endothelin-1, E-selectin, and vascular cell adhesion molecule-1 (VCAM-1) in controls and CSX patients were measured, both at the baseline and after the treatment, by the enzyme-linked immunosorbent assay. In CSX patients, at the baseline, levels of E-selectin and VCAM-1 were significantly higher than those of the controls. In addition, levels of these biomarkers in CSX patients after the treatment significantly decreased compared to the baseline. In spite of similar tendency, these differences were not significant for endothelin-1. In conclusion, metoprolol therapy improves endothelial function. Thus, it may be a suggested choice for CSX treatment. However, further studies are needed to confirm the clinical significance of metoprolol therapy for CSX patients.

## Introduction

Endothelium has an important role in the regulation of vascular tone, cellular adhesion, thrombo resistance, and vessel wall inflammation. Endothelium regulates vasomotion, not only by releasing vasodilator substances, but also by elevating constrictor tone via the generation of endothelin and vasoconstrictor prostanoids as well as conversion of angiotensin I into angiotensin II at the endothelial surface (1).

The term endothelial dysfunction is most often used to denote the impairment of endothelium-dependent vasodilation; but, it probably includes those conditions leading to endothelial activation with abnormalities in endothelial interactions with leukocytes, platelets, and regulatory substances (2-5). 

Evidence supporting endothelial dysfunction as the cause of cardiac syndrome X (CSX; angina-like chest pain despite normal coronary angiogram) has been derived from the studies assessing coronary flow response to vasoactive stimuli(6). Studies have shown the impairment of both endothelium-dependent and -independent coronary vasodilation(7, 8) 

In addition, as a result of endothelial dysfunction, CSX patients have significantly reduced plasma NO level in comparison to control subjects. On the other hand, vasoconstrictor peptide endothelin -1 (ET-1) which is a marker of endothelial damage has been increased in patients with CSX; so, it is hypothesized that elevated ET-1 levels might be involved in the development of micro-vascular dysfunction in CSX patients. Accordingly, the imbalance between NO and endothelial markers has been recently found to be associated with reduced coronary vasomotor responses in CSX patients. 

The important factor for the treatment of CSX is improvement in endothelial function(9, 10). Although many different traditional agents such as statins, angiotensin converting enzyme (ACE) inhibitors, and nitrates have been used in the treatment of CSX, the most effective and appropriate medication has not been clearly proven(11, 12). 

Metoprolol, a beta-blocker, is a first-generation cardio-selective adrenoceptor antagonist and has been demonstrated to be effective in reducing the risk of morbidity and mortality associated with cardiovascular diseases such as heart failure and age-related diseases characterized by endothelial dysfunction (13). Many studies have shown that metoprolol not only increases the ratio of bioavailable NO to peroxynitrite, but also increases the rate of NO generation and decreases rate of NO decay (14, 15). In the present study, endothelial function was investigated in these patients by measuring three important endothelial markers including ET-1, E-selectin, and vascular cell adhesion molecule-1 (VCAM-1) and assessing effects of metoprolol therapy on these parameters and as a result on endothelial function.

## Experimental

*Patients with cardiac syndrome X*

Twenty-five consecutive patients (20 female / 5 male, mean age of 55.36 ± 10.31 years) who were referred to Department of Cardiology Urmia University of Medical Sciences, Urmia, Iran; for suspected coronary artery diseases were enrolled in this case-control study. Also, data was collected before and after the intervention.

Inclusion criteria were the history of stable effort-related angina, a normal 12-lead ECG at rest, positive exercise tests for ischemia-like ECG changes, normal left and right ventricular functions at rest, and normal coronary angiogram. Exclusion criteria were heart structural and functional diseases such as valvular heart disease and myocardial hypertrophy on echocardiography, other systemic diseases like diabetes mellitus, any active infective diseases, known connective tissue diseases, pulmonary, renal, hepatic, or hematological disorders, and no concomitant acute and chronic diseases. None of the patients were taking substitutive vasoactive agents such as angiotensin-converting enzyme inhibitors, calcium channel blockers or β-blockers. All the medical history regarding the presented complaints, comorbidities, risk factors, and used medications was collected prior to the physical examination in a quiet and temperature-controlled room (22 °C) before noon.

After the enrollment of patients, 7 mL heparin tube containing blood sample was obtained from each subject and centrifuged at 2000× *g* for 15 min. Plasma was aliquoted and stored at -80 ° C until analysis. Then, metoprolol (50 mg BID) was orally given daily to the subjects and continued for 4 weeks. After the mentioned time, the plasma samples were collected in the same condition and stored for biochemical analysis.

*Control group*

Age- and sex-matched healthy volunteers (20 female /5 male, mean age: 54.32 ± 9.27 years) who were recruited from among the people coming to the laboratory for checking up and examination results were served as the controls. Furthermore, they had no concomitant acute or chronic diseases and did not take any kinds of medication or dietary supplements, including vitamins, antioxidants, and so on.

All subjects gave their informed consent prior to their inclusion in the study. The study protocol approved by the ethical guidelines of the 1975 Declaration of Helsinki as reflected in the guidelines of the Medical Ethics Committee, Ministry of Health, Iran; by our university Medical Entices committee as UMSU-91/3/2-86 protocol.

*Biochemical analysis*

Plasma samples were used for markers. For E-selectin measurement, sandwich ELISA method and commercial kit (BOSTER immunoleader, China) with the sensitivity of <4 pg/mL were used. A similar method was used for endothelin-1 (Glory Science- USA; sensitivity of 1.25 ng/L) and VCAM-1 (BOSTER immunoleader, China; sensitivity of < 10pg/mL). All the three markers were measured in three groups of control, baseline, and after the treatment.

*Statistic analysis*

For qualitative parameters such as sex and quantitative ones such as age, body mass index , blood pressure, and endothelial markers, proportion and mean± standard deviation were used, respectively. Kolmogorov–Smirnov test was applied to determine the continuous variables were normally distributed or not. For comparing quantitative parameters, for parameters with unmoral distribution, and for comparing before and after treatment groups, t-test and Mann- Whitney test were used, respectively. P < 0.05 was accepted as statistically significant and SPSS 16 was used for statistical analysis.

## Results

All the patients and controls completed the study. The main demographic characteristics of all the groups are shown in Table 1. The first part of this study was the comparison of endothelial markers levels between the controls and CSX patients before the treatment. Levels of E-selectin and VCAM-1 in CSX patients were significantly higher than those in the controls (p = 0.010 and p = 0.002, respectively, Table 2.). Difference of ET-1 levels was not significant (p = 0.332, Table 2.).

In addition, the next part of the study was about assessing endothelial biomarkers levels in CSX patients before and after the using metoprolol therapy. In this part, only levels of VCAM-1 (p = 0.006) were found to be significantly lower in patients after the treatment compared to those before the treatment. Also, in this study, the measured levels of endothelin and E-selectin in CSX patients after treatment were tended to be lower than CSX patients at baseline, but they were greater than controls (Table 2.).

**Table 1:** Demographic characteristics of cardiac syndrome X (CSX; n=25) and control (n=25) group

	**CSX group**	**Control group**	**P value**
Age (years) *	55.3610.31	54.329.27	0.654
Sex (male/female)	5/20	5/20	1.000
Body masss index (Kg/m2) *	30.164.23	26.334.14	0.001
Systolic blood pressure (mmHg) *	12414.43	122.404.35	0.861
Diastolic blood pressure (mmHg) *	78.807.25	78.805.25	0.862

* meanSD for age, body mass index, Systolic blood pressure and Dyastolic blood pressure

**Table 2.** Endothelial biomarkers levels in controls (n=25) and CSX patients before and after treatment (n=25) with metoprolol (50 mg BID).

**Variable**	**Controls**	**CSX** **before treatment**	**CSX** **after treatment**	**P** *	**P** *	**P** ***
E-selectin (ng/mL) *	9.294.77	18.7812.64	14.857.75	0.010	0.001	0.055
Endothelin (ng/mL) *	124.5853.78	143.38103.09	131.7110.81	0.332	0.778	0.657
VCAM-1 (ng/mL) *	57.1217.42	109.0848.21	84.6330.88	0.002	<0.001	0.006

MeanSD for E-selectin, Endothelin-1 and vascular cell adhesion molecule-1 (VCAM-1)

* P= control *vs*. CSX before treatment;

**= P control *vs*. CSX after treatment;

*** P= CSX before treatment *vs.* CSX after treatment.

## Discussion

Cardiac syndrome X is a controversial subject because of its identity, mechanisms, pathogenesis, prevalence, and clinical importance. Many recent studies have introduced endothelial function as an important pathological mechanism for CSX (10). Endothelial function may be evaluated based on the biochemical assessment of the quantities of endogenous substances released from endothelial cells (16).

In many studies, inflammation in micro-vascular systems has been one of the important causes of endothelial dysfunction. For instance, Senen *et al*., (17) assessed two adhesion molecules including E- selectin and P-selectin in CSX patients and found higher levels of these molecules in CSX patients. They concluded that it reflected chronic inflammation and endothelial dysfunction. In the study designed by Tousoulis *et al*., (18) levels of VCAM-1 and ICAM-1 were measured in CSX patients and compared with healthy controls. Results demonstrated that levels of these molecules were significantly higher in CSX patients than controls. In this study, E-selectin and VCAM-1 levels were compared in CSX and controls. The finding was consistent with Senen *et al*., (17) and Tousoulis *et al*., (18) results. In summary, inflammation and increased inflammatory factors could be said to cause endothelial dysfunction and finally low blood and oxygen supply to some areas of heart manifested as CSX. Kaski *et al*., measured ET-1 levels as an important modulator of micro-vascular function in CSX patients and found that its levels were significantly higher in CSX patients than controls. They concluded that the association between «high» plasma ET-1 and an earlier onset of chest pain during exercising could suggest that ET-1 may also have a role in the genesis of chest pain in patients with normal coronary arteries(10). In addition, in another study, Kocak *et al*., measured ET-1 levels in CSX patients and controls at rest and during exercising in order to investigate endothelial function in these patients. They found that ET-1 levels of patients were significantly higher than those of controls. So, they deduced that increased levels of ET-1 may have a role in the pathogenesis of this syndrome (19). 

These findings were in agreement with those of the present study, in which ET-1 levels were higher in CSX patients than controls; however, it was not significant in our study. 

Furthermore, management of patients with CSX is a complex challenge. Lack of a unique definition for it, varied pathogenic mechanisms responsible for the condition, and different diagnostic criteria used by different investigators have contributed to the complicated management of these patients. Nevertheless, beta-blocker therapy may be considered the first line of treatment for CSX patients. Recently, many studies have investigated beta-blockers for getting new information. For example, in a study done by Fragasso *et al.,* (20), both atenolol and propanolol were shown to improve daily-life angina, ST segment depression during continuous electrocardiographic monitoring, exercise-induced ST segment changes, and left ventricular diastolic function.

Generally, endothelial protective effects of beta-blockers are weak known. However, the involvement of NO in the vasodialtory mechanism of action of some beta-blockers has been recently understood. An elevation of intracellular calcium by the activation of phospholipase C and increase of NO release on the estrogen receptors on endothelial cells have been postulated as the mechanism of NO release by nebivolol (21). 

Moreover, Bank *et al.*, investigated effects of carvedilol versus metoprolol on endothelial function in 31 diabetic patients. They measured C-reactive protein and di-methyl arginine levels and assessed endothelial function by flow-mediated dilation (FMD). The conclusion was that carvedilol had better effects on endothelial function (22). 

In the studies that have been performed thus far, effects of metoprolol on endothelial function have not been investigated by measuring endothelial markers in CSX patients. In the present study, levels of all three markers including E-selectin, ET-1 and VCAM-1 decreased in all the patients after the four-week treatment, representing that metoprolol may improve endothelial function or inhibit endothelial dysfunction in CSX patients.

## Conclusion

In summary, it seems that metoprolol may be a suggested choice for CSX treatment. So, it can be concluded that improvement of endothelial function is one of the treatment lines for CSX patients. But, for obtaining the best results, new investigations must be performed.
